# The Use of Matriderm^®^ for Scalp Full-Thickness Defects Reconstruction: A Case Series

**DOI:** 10.3390/jcm11206041

**Published:** 2022-10-13

**Authors:** Giovanni Dell’Aversana Orabona, Francesco Maffia, Giovanni Audino, Vincenzo Abbate, Cristiana Germano, Paola Bonavolontà, Antonio Romano, Riccardo Villari, Mauro Mormile, Luigi Califano

**Affiliations:** 1 Maxillofacial Surgery Unit, Department of Neurosciences, Reproductive and Odontostomatological Sciences, University Federico II, Via Pansini 5, 80100 Naples, Italy; 2 Infectious Diseases Unit, Department of Clinical Medicine and Surgery, University of Naples Federico II, 80131 Naples, Italy; 3 Pneumology Unit, Department of Clinical Medicine and Surgery, University of Naples Federico II, 80131 Naples, Italy

**Keywords:** scalp defects, skin malignancies, Matriderm^®^ dermal substitute, reconstructive surgery, plastic surgery

## Abstract

Background: The scalp region represent a common area affected by benignant and malignant skin tumor, and it represents a surgical challenge when it is needed to be reconstructed. The aim of this study is to present our experience with full-thickness scalp skin defects, reconstructed using Matriderm^®^ dermal substitute and split-thickness skin graft (STSG). Methods: A retrospective analysis of patients treated for scalp region reconstruction was conducted with 16 patients. All patients underwent the same procedure: scalp full-thickness tumor excision with simultaneous reconstruction with Matriderm^®^ and the application of a split-thickness skin graft in the same surgical time. During follow-ups, the surgical outcome was evaluated by accurate clinical examination of the wound, adopting the Vancouver Scar Scale (VSS). Results: The outcomes obtained were satisfying: wound healing at the end of the procedures was optimal, grafted skin resulted similar to surrounding tissue, and pigmentation and vascularity showed a decrease in the period between 6 months and 1 follow-up. Conclusions: The use of Matriderm^®^ and split-thickness skin grafting for scalp full-thickness defects reconstruction resulted in an optimal, stable, and safe procedure, suitable for elderly patients.

## 1. Introduction

The reconstruction of full-thickness skin defects of the scalp represents a challenge for the maxillofacial surgeon [1]. The scalp region represents a common area affected by benignant and malignant skin tumor, especially in older patients [2]. Considering the behavior of the malignant tumors of the skin, the difficulty to obtain clear margins should be considered to avoid unappropriated reconstruction that could bury an eventual recurrence under a flap [3]. Usually, these pathologies are described in an older patient affected by comorbidities that increase the intolerance of major reconstructive surgery [4]. The surgical options described in the literature are: full-thickness skin grafts (FTSGs), split-thickness skin grafts (SPTSGs), dermal substitute combined with single/delayed time SPSTGs, and, in restricted cases, locoregional flaps [5]. The target of these reconstructive techniques is trying to restore the normal skin-layered structure, in particular, the dermal layer [4]. In the literature, the adoption of dermal substitutes with skin grafting in a two steps surgical procedure is offering promising results in the reconstruction of full-thickness scalp defects [3]. The procedure is simple and fast, gives the possibility to control the disease and reduces the risk of infection. The aim of this study is to present our experience with full-thickness scalp skin defects, reconstructed using Matriderm^®^ dermal substitute (Dr. Suwelack Skin & Health Care AG, Billerbeck, Germany) and split-thickness skin graft (STSG).

## 2. Materials and Methods

### 2.1. Preoperative Evaluation

A retrospective analysis of patients treated for scalp region reconstruction in the Oral and Maxillo-Facial Department of the “Federico II” University of Naples was conducted during the period between 2017 and 2021. Inclusion criteria for the selected study were tumor excision in scalp region with full-thickness defect, implantation of Matriderm^®^ dermal substitute, 1 year of follow-up. Patient data were collected from the department database, including age, sex, comorbidities, surgical procedures, histopathological examination, and follow-up records.

### 2.2. Surgical Procedure

**Figure 1:**
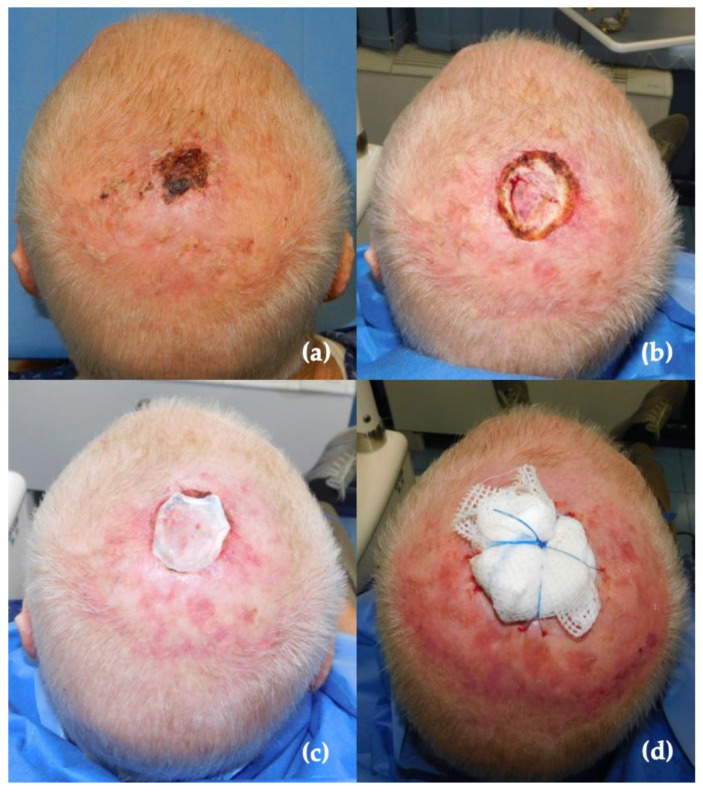
(**a**) Preoperative presentation of the scalp skin malignancy (SCC). (**b**) Full-thickness excision of the lesion. (**c**) Matriderm implantation on the wound bed. (**d**) Final packaging of the reconstruction with greasy gauzes.

Every patient underwent the same surgical procedure performed by the same surgical team: scalp full-thickness tumor excision with simultaneous reconstruction with Matriderm^®^, and the application of a split-thickness skin graft in the same surgical time (Figure 1a–d). The excision was performed with 1 cm of margin from the considered lesion. Once excised, the Matriderm^®^ sheet was shaped to perfectly fit the surgical gap and then soaked with 0.9% saline solution. Matriderm^®^ was anchored to surgical edges by Vicryl 3.0 stitches and then covered with a whole or meshed split-thickness skin graft, taken from the thigh. The bilayer reconstruction was then covered and compressed with a sterile greasy gauzes package. In the most clinically suspicious cases, the technique was divided into two steps in order to reconstruct with an STSG and Matriderm^®^, only once the margins were certain.

### 2.3. Postoperative Evaluation

During follow-ups, the surgical outcome was evaluated by accurate clinical examination of the wound adopting the Vancouver Scar Scale (VSS), considering: pigmentation (from 0 to 2 points), vascularity (0 to 3 points), pliability (0 to 5 points), height (0 to 3 points). The lower the score, the better the scar status.

## 3. Results

**Figure 2:**
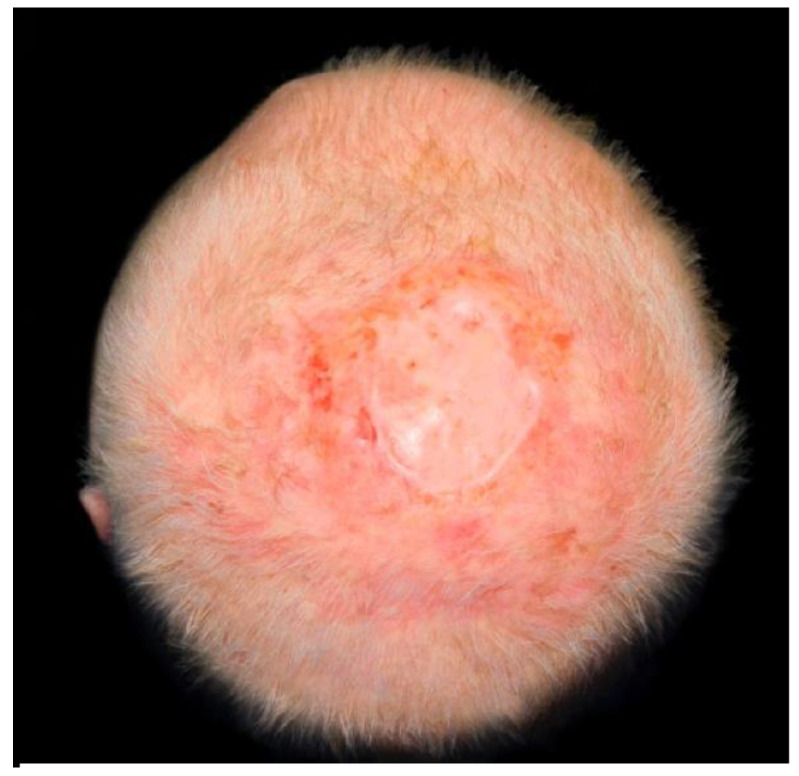
Final scar result at 12 months follow-up.

At the end of the first analysis, a total of 47 patients treated for scalp region reconstruction were considered. Thirty-one patients were excluded for different reasons: no Matriderm^®^ use, split-thickness defects, full-thickness defects closed by local flaps, reconstruction not related to tumor excision but locoregional flap harvesting. Sixteen patients were conclusively selected and enrolled in the study. All enrolled patients filled in written informed consent for the study. The mean age of patients was 75 years old, with a range from 68 to 94. The ratio between males and females was 3:1. A total of 62.5% of patients (10/16) were treated with local anesthesia, while six patients (37.5%) underwent general anesthesia. Only two patients were treated with a two steps technique, and both margins were clear. The shape of the full-thickness defect was approximately round, with an average maximum diameter of about 5.86 cm and with a range from 4.5 cm to 8.3 cm. The most frequent histopathologic finding was basal cell carcinoma (10/16) with 62.5% of cases, followed by squamous cell carcinoma (6/16, 37.5%). Among the SCC cases, two of them were reported as actinic keratosis with SCC in situ. Follow-ups were recorded at 1 month, 6 months, and 1 year. Clinical follow-ups showed good recovery in the majority of patients (Figure 2). Two patients reported positive surgical margins and underwent a second surgery to enlarge the excision. One patient experienced surgical infection during the first wound healing; 10 days after the first procedure, a wound curettage was performed and Matriderm^®^ matrix was removed and substituted. Two weeks after the curettage, a split-thickness graft was sutured upon the wound. An improvement in the scar status during the recorded follow-ups was observed: the VSS score at 6 months was 4875 with a range between 3 and 6. The mean VSS score at 1 year was 16,875 with a range between 0 and 2. The highest improvements were observed in pigmentation and vascularity, while height remained mostly unchanged. Postoperative alopecia occurred in the reconstructed region, with acceptable aesthetic outcomes considering that most of the patients were bald.

## 4. Discussion

The choice of the appropriate technique for the reconstruction of full-thickness skin defects usually depends on the depth and size of the defects [4]. Local flaps of the free flap are the most suggested procedure but require a good patient status to ensure the wanted outcome [6]. To bypass these limits, the adoption of STSGs and FTSGs should be considered. The use of FTSGs is limited to small defects, offering less contraction and optimal aesthetical outcomes [1]. SPTSGs are adopted for larger defects, but scar contraction and poor dermal layer transfer can increase the risk of complications [5]. Both techniques must consider the donor site comorbidity and the need for a well-vascularized wound bed to ensure proper healing [1]. The use of just skin grafts is a valid option but when noble structures, such as bone, nerves, and tendons, are exposed it should be avoided due to the high risk of failure [7].

A diffused technique is to adopt a dermal substitute in the first layer of the reconstruction, followed by a second layer where an STSG is positioned above the granulating wound [5,7]. In this way, the dermal restoration given by the dermal substituteallowed a better healing of the future skin graft [5,8]. The procedure can also be divided in a two-step procedure, with a shot amount of time between dermal apposition and SPTSGs application: in this way, there is the possibility to re-operate effortlessly in case of positive margins or visible recurrence. Furthermore, this avoids the burying of the eventual tumor recurrence under a single-time FTSG [3]. Many dermal substitutes are now available on the market, the most used are Matriderm^®^, Alloderm^®^ (Lifecell Corporation; Branchburg, NJ, USA), and Integra^®^ (Integra, Integra LifeSciences Corporation; Princeton, NJ, USA) [4,9]. The Integra^®^ substitute is bilayered and it acts differently; the outer layer is made by silicone that simulates the epidermis and must be removed 3 weeks after the application, while the inner layer is the dermal matrix.

In our case, the choice was Matriderm^®^, a highly porous membrane based on freeze-dried acellular collagen with a thickness of 1 or 2 mm as a dermal prosthesis. It is composed of type I, III, and V bovine dermal collagen and hydroxylated elastin [10]. Dermal substitutes act as a collagen scaffold for fibroblast, promoting neovascularization and rapid cell migration [11]. The histological phases of dermal regeneration following dermal substitute implantation are fourfold: scaffold imbibition, fibroblast migration, neovascularization, and remodeling with final maturation [12]. Matriderm^®^ resorbs in 6 weeks, after collagen production via fibroblasts [2].

The adoption of dermal substitutes in the treatment of large wounds, burns, and multilayer reconstructions is widely diffused in the literature. Considering, in particular, the reconstruction of scalp defects, it is well established that the combination of dermal substitutes and STSGs offers a more durable and thicker coverage than STSGs only procedure, giving better aesthetical and functional outcomes [3,5]. In our population, the outcomes obtained were satisfying: wound healing at the end of the procedures was optimal, grafted skin resulted similarly to the surrounding tissue, and pigmentation and vascularity showed a decrease in the period between 6 months and 1 follow-up. All these elements suggest how the procedure adopted allows long-term stability and protection of the graft, with a lower rate of complications, such as infections or seromas. From the organizational point of view, operative time was shorter if compared with major surgery as locoregional flaps and also patients’ hospitalization was reduced or not even necessary. The patients treated in the day surgery regimen came back weekly for medications. The major difficulty in the reconstruction of scalp region losses of substance is represented by the cranial theca exposure: bone exposition can mine the success of the procedure due to the restricted vascularized bed [7]. For this reason, a multilayer reconstruction implanting a dermal substitute miming a vascularized tissue on the exposed fragile structure offers proper wound healing [1,13]. In larger defects, the outer table of the cranium can be drilled to increase the matrix imbibition [12].

Based on our experience and what is reported in the literature, the only disadvantages of this procedure are represented by the cost of the membrane and its thickness sometimes not satisfactorily filling the gap height [14]. In fact, considering the results of the VSS, the wound height represented the value with the lower bettering, due to the presence of a millimetric gap between the surrounding skin and the reconstructed one. In the literature, the application of VSS for the evaluation of the scar following the use of dermal substitute and STSG has been described for donor site closure after radial forearm free flap harvesting [8]. Considering the locoregional anatomy discrepancy, our results were comparable to the ones obtained in these studies. To the best of our knowledge, this is the first study to apply VSS to scalp wounds.

## 5. Conclusions

Reconstructive surgery must consider benefits and disadvantages to offer a reasoned solution adapted to the patient. The use of Matriderm^®^ and split-thickness skin grafting for scalp full-thickness defects reconstruction resulted in an optimal, stable, and safe procedure, suitable for elderly patients. The availability of dermal matrices has created the possibility of overcoming important surgical limitations, such as bone exposure in large defects, suboptimal aesthetic results, and residual scarring. The possibility of obtaining promising results with a simple, effective, and safe intervention makes dermal substitute implantation the first choice for scalp full-thickness defects reconstruction.

## Data Availability

The data was acquired from our patient archives.
